# Author Correction: Organophosphorus diisopropylfluorophosphate (DFP) intoxication in zebrafish larvae causes behavioral defects, neuronal hyperexcitation and neuronal death

**DOI:** 10.1038/s41598-021-85547-1

**Published:** 2021-03-09

**Authors:** Alexandre Brenet, Julie Somkhit, Rahma Hassan-Abdi, Constantin Yanicostas, Christiane Romain, Olivier Bar, Alexandre Igert, Dominique Saurat, Nicolas Taudon, Gregory Dal-Bo, Florian Nachon, Nina Dupuis, Nadia Soussi-Yanicostas

**Affiliations:** 1 NeuroDiderot, Inserm, Université de Paris, 75019 Paris, France; 2 Département de Toxicologie Et Risques Chimiques, Institut de Recherche Biomédicale Des Armées (IRBA), 91 220 Brétigny‑sur‑Orge, France; 3 Institut de Recherche Biomédicale Des Armées (IRBA), Unité de Développements Analytiques Et Bioanalyse, 91 220 Brétigny‑sur‑Orge, France

Correction to: *Scientific Reports* 10.1038/s41598-020-76056-8, published online 05 November 2020

The Acknowledgements section in this Article was omitted. The Acknowledgements section should read:

“J.S. was supported by a doctoral fellowship from the Direction Générale de l’Armement (DGA, 2018, 60 0059).”

