# Supplementary material for: Organophosphorus diisopropylfluorophosphate (DFP) intoxication in zebrafish larvae causes behavioral defects, neuronal hyperexcitation and neuronal death
Source: Sci Rep. 2020 Nov 5;10:19228. doi: 10.1038/s41598-020-76056-8 (PMC7645799; doi:10.1038/s41598-020-76056-8)
Supplement: Supplementary file 1 — Supplementary Information 1. [file 41598_2020_76056_MOESM1_ESM.docx]

**Organophosphorus diisopropylfluorophosphate (DFP) intoxication in zebrafish larvae causes behavioral defects, neuronal hyperexcitation and neuronal death**

*Alexandre Brenet^1†^, Julie Somkhit^1†^, Rahma Hassan-Abdi^1^, Constantin Yanicostas^1^, Christiane Romain^1^, Olivier Bar^1^, Alexandre Igert^2^, Dominique Saurat^3^, Nicolas Taudon^3^, Gregory Dal-Bo^2^, Florian Nachon^2^, Nina Dupuis^2#^, and Nadia Soussi-Yanicostas^1#^**

^1^ Université de Paris, NeuroDiderot, Inserm, F-75019 Paris, France

^2^ Institut de Recherche Biomédicale des Armées (IRBA), Département de toxicologie et risques chimiques, F-91 220 Brétigny-sur-Orge, France.

^3^ Institut de Recherche Biomédicale des Armées (IRBA), Unité de développements analytiques et bioanalyse, F-91 220 Brétigny-sur-Orge, France.

Co-first (†) and (#) co-last authors

* Correspondence: nadia.soussi@inserm.fr

**Supplementary Materials and methods**

**Measuring DFP stability**

A 15 µM solution of DFP was prepared following dilution in fish water containing 1% DMSO, and 200 µL water samples were removed at different incubation times (0, 2, 4, 6 and 24 h; *n* = 3 per condition) and were stored at 20 °C until extraction. 360 µL of ethyl acetate (VWR) was added to 180 µL of the sample, and after vigorous shaking, aliquots (100 µL) of organic phases were recovered and stored at 4 °C until analysis by gas chromatography-mass spectrometry (GC-MS). Residual DFP concentrations were determined by GC-MS analyses conducted with a gas chromatograph (Agilent 6890N) coupled to a quadrupole mass spectrometer equipped with an EI source (Agilent MSD5973). After pulsed splitless injection (40 psi) at 220 °C, the GC separation was performed on a Rtx-OPP2 column (Restek, 30 m × 0.25 mm × 0.25 µm) using a linear ramp from 40 °C to 280 °C (20 °C/min). The mobile phase was helium (99.9995%) at a flow rate of 1.2 mL/min. The source and quadrupole temperatures were set at 230 °C and 150 °C. Acquisition was performed in the SIM (single ion monitoring) mode (*m/z* 101 and 127 as quantifier and qualifier ions). Operating software was MassHunter Workstation Quantitative analysis version B.09.00/Build 9.0.647.0. DFP concentrations were calculated based on a linear calibration curve (individual residuals within ± 20%), obtained with solutions of DFP diluted in ethyl acetate and ranging from 0.05 to 1.35 µg/mL.

**Hematoxylin/eosin staining**

5 dpf larvae exposed to either DFP or vehicle (1% DMSO) were anesthetized using 0.01% tricaine, fixed with 10% formaldehyde, paraffin-embedded and sectioned. Sections were deparaffinized and rehydrated before hematoxylin and eosin staining. Freshly stained sections were treated with ethanol and xylene, and then mounted in Pertex medium. Sections were imaged using a Nikon Eclipse microscope (E-200) equipped with a digital sight (Nikon).

**AChE activity**

5 dpf zebrafish larvae (20 larvae per sample) were collected 2, 4, and 6 h after DFP exposure, and stored at -80 °C until further analysis. Samples were homogenized in 50 mM phosphate buffer, 0.5% Tween 20 (pH 7.4) using a Precellys homogenizer and 1.4 mm ceramic beads, centrifuged at 10,000 × *g* (4 °C) for 10 min, and the supernatants were collected and stored at --80 °C. Total protein concentration was determined using the DC Protein Assay (Bio-Rad) and all samples were diluted to 1.2 mg/ml. AChE activity was measured by adding 1 mM acetylthiocholine (Sigma) and 0.22 mM 5,5′-dithiobis-2-nitrobenzoic acid (DTNB, Sigma) to the samples, which were then incubated at 25 °C. The compound made as the result of the reaction of thiocholine and DTNB was monitored for 30 min with a spectrometer at 412 nm equipped with a microplate reader. All the samples were assayed in duplicate. The final results were expressed as percentages of average control activity.

**Activated-caspase-3 immunolabelling**

Five dpf larvae treated with either DMSO or DFP were fixed with 4% formaldehyde for 1h30. Brains were then dissected and immunostaining was performed. Briefly, brains were washed first with 0.1% then 1% triton in PBS, permeabilized and blocked with 2% triton, 1% DMSO, 5% Goat serum in PBS for 2 hours. Brains were incubated overnight with rabbit polyclonal activated-caspase-3 antibody (Abcam, Ab44976, 1:500). Next-day, brains were washed with 0.1% triton in PBS several times and were incubated for 1h with anti-rabbit Alexa 488 conjugated secondary antibody (Molecular Probes, A-21206 1:300). Finally brain were washed one last time with 0.1% triton before agar-embedded and imaged with a Leica SP8 laser scanning confocal microscope equipped with a 20x/0.75 multi-immersion objective.

**Supplementary table 1: qRT-PCR Primer**

| Genes | GenBank accession number | Forward Primer | Reverse Primer |
| --- | --- | --- | --- |
| *fosab* | ENSDARG00000031683 | 5’-AAC CAG ACT CAG GAG TTC AC-3’ | 5’-GGA GAA AGC TGT TCA GAT CTG-3’ |
| *atf3* | ENSDARG00000007823 | 5’-GAT GAA GCG AGA GGC TGT C-3’ | 5’-TCA GCT CTG CAT TGA TGG AC-3’ |
| *junB* | ENSDARG00000074378 | 5’-CAC CTG TCG AAT TGA CGC TG-3 | 5’-GAT GAC GCC GTT ACC GTT C-3’ |
| *npas4a* | ENSDARG00000055752 | 5’-CAA GAT ATA ACT ACA GCT AGC AG-3’ | 5’-GAG CGA CCA AAT CTA CCA TTG-3’ |
| *npas4b* | ENSDARG00000087753 | 5’-CAT TCC ATG GTG GAT CTA GTG-3’ | 5’-AAA GAG GCG GTC TGT GTC AG-3’ |
| tbp | ENSDARG00000014994 | 5’-GTG CAC AGG AGC CAA AAG TG-3’ | 5’-GTT CAT AGC TGC TAA ACT GCT G-3’ |

**Supplementary table 2: List of antibodies used for immunofluorescence studies**

| **Antibodies** | **Primary/**  **Secondary** | **Supplier** | **Species** | **Type** | **Dilution** | **Reference** |
| --- | --- | --- | --- | --- | --- | --- |
| C-fos | Primary | Santa Cruz Biotechnology | Rabbit | Polyclonal | 1/200 |  |
| GAD56-67 | Primary | Chemicon | Rabbit | Polyclonal | 1/500 | ab1511 |
| Gephyrin | Primary | Abcam | Rabbit | Polyclonal | 1/100 | ab32206 |
| NMDAR2B | Primary | Abcam | Rabbit | Polyclonal | 1/500 | ab71178 |
| Caspase-3 | Primary | Abcam | Rabbit | Polyclonal | 1/500 | Ab44976 |
| Biotinylated goat anti-rabbit antibodies | Secondary | Vector, Burlingame, CA | Goat | Secondary Antibody | 1/200 | BA‐1000 |
| Anti-Rabbit IgG (H+L)-Alexa 488 | Secondary | Molecular Probes | Goat | Secondary Antibody | 1/500 | A-1134 |
