# Supplementary material for: Organophosphorus diisopropylfluorophosphate (DFP) intoxication in zebrafish larvae causes behavioral defects, neuronal hyperexcitation and neuronal death
Source: Sci Rep. 2020 Nov 5;10:19228. doi: 10.1038/s41598-020-76056-8 (PMC7645799; doi:10.1038/s41598-020-76056-8)
Supplement: Supplementary file 2 — Supplementary Information 2. [file 41598_2020_76056_MOESM2_ESM.pdf]

Organophosphorus diisopropylfluorophosphate (DFP) intoxication in zebrafish larvae causes behavioral defects, neuronal hyperexcitation and neuronal death

*Alexandre Brenet[1†], Julie Somkhit[1†], Rahma Hassan-Abdi[1], Constantin Yanicostas[1], Christiane Romain[1], Olivier Bar[1], Alexandre Igert[2], Dominique Saurat[3], Nicolas Taudon[3], Gregory Dal-Bo[2], Florian Nachon[2], Nina Dupuis[2#], and Nadia Soussi-Yanicostas[1#]**

[1] Université de Paris, NeuroDiderot, Inserm, F-75019 Paris, France
[2] Institut de Recherche Biomédicale des Armées (IRBA), Département de toxicologie et risques chimiques, F-91 220 Brétigny-sur-Orge, France.
[3] Institut de Recherche Biomédicale des Armées (IRBA), Unité de développements analytiques et bioanalyse, F-91 220 Brétigny-sur-Orge, France.

Co-first (†) and co-last (#) authors

* Correspondence: nadia.soussi@inserm.fr

Supplementary figures

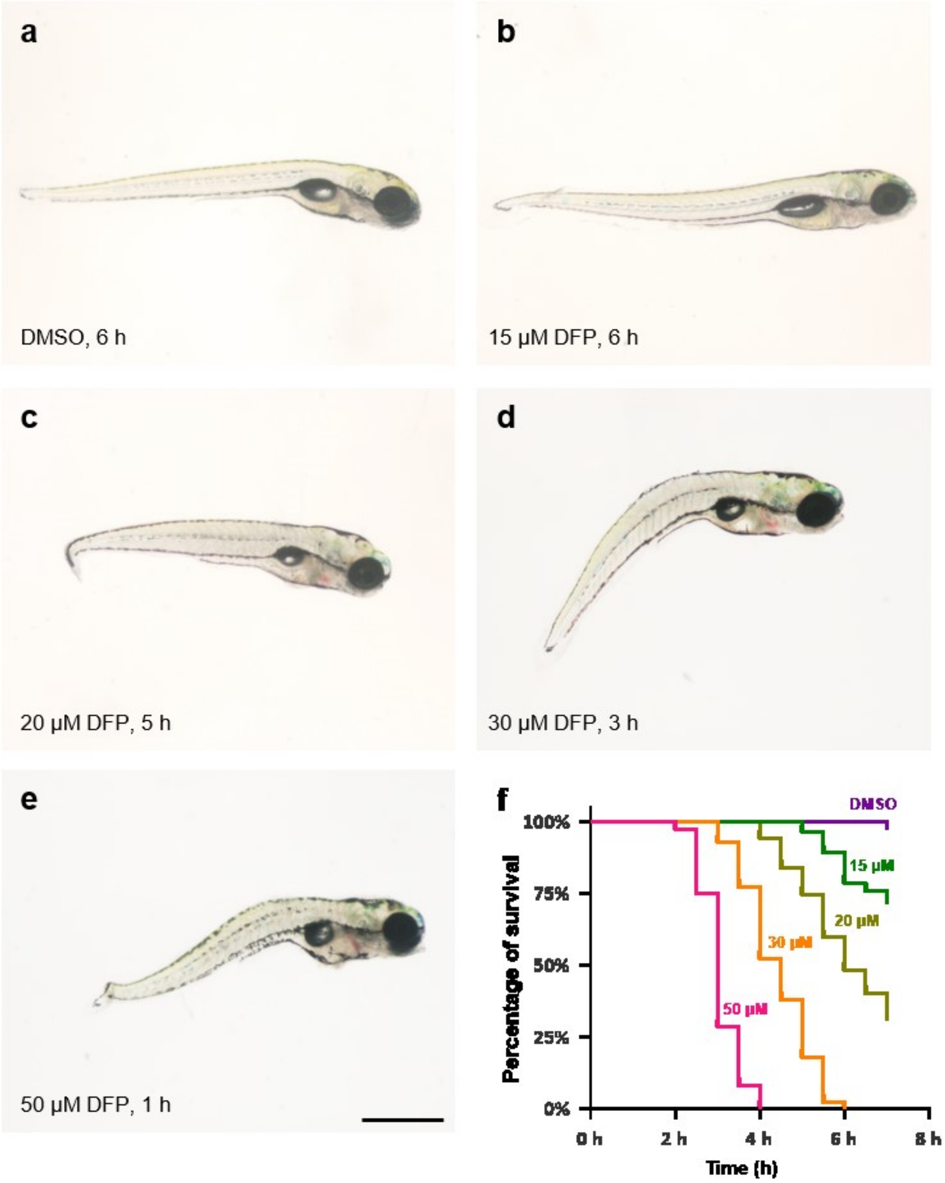

Supplementary Fig. 1. DFP exposure caused phenotypic defects and larval lethality. (**a**-**e**) Phenotypes of 5 dpf larvae exposed to either vehicle (DMSO) for 6 h (**a**), or 15 µM DFP for 6 h (**b**), or 20 µM DFP for 5 h (**c**), or 30 µM DFP for 3 h (**d**), or 50 µM for 1 h (**e**). Scale bar: 1 mm. (**f**) Survival curve of 5 dpf larvae exposed to either vehicle (DMSO) or 15, 20, 30 or 50 µM DFP.

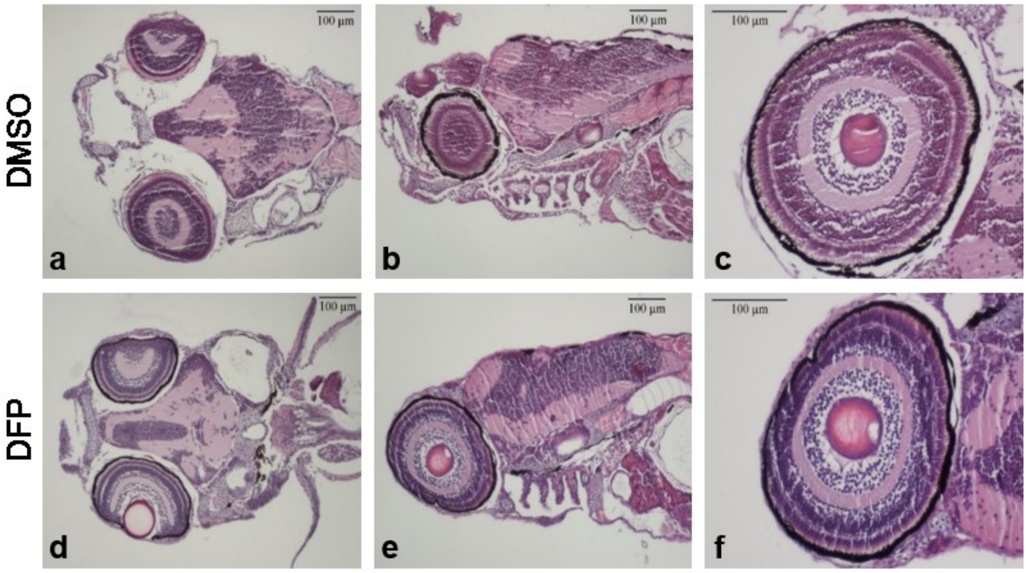

Supplementary Fig. 2. Zebrafish larvae exposed for 6 h to 15 μM DFP do not show visible phenotypic defects. (**a-f**) Horizontal (**a, d**) and sagittal (**b, c, e, f**) tissue sections of 5 dpf larvae (**a, b, d, e**) and corresponding eyes (**c, f**), following exposure for 6 h to either vehicle (DMSO) (**a, b, c**) or 15 μM DFP (**d, e, f**)

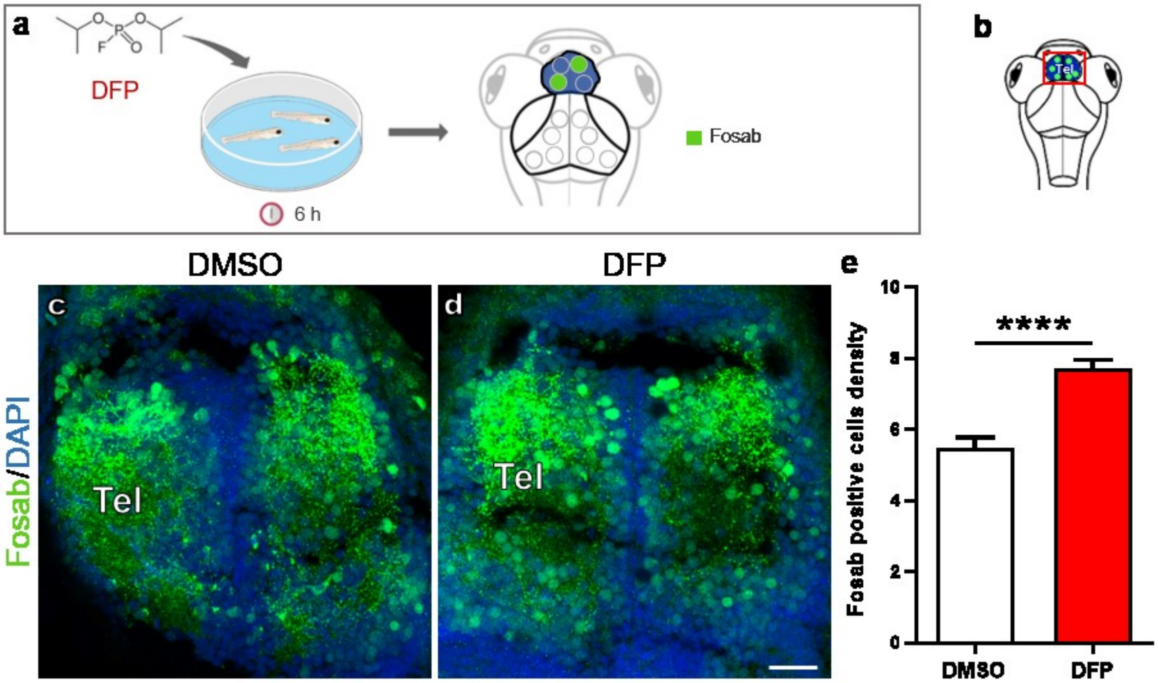

Supplementary Fig. 3 DFP exposure increase Fosab protein and gene expression. (**a**) In the experimental set-up, 5 dpf larvae were exposed to either 15 µM DFP or vehicle (DMSO) for 6 hours, prior to being processed for either Fosab immunostaining or neuronal activity related gene expression. (**b**) Scheme of 5 dpf larvae head with the red box showing the regions of interest in the brain. (**c**, **d**) Fosab immunolabelling of neurons in the telencephalon of 5 dpf larvae exposed to DMSO (**c**) or 15 µM DFP (**d**). Scale bar: 20 µm. (**e**) Quantification of the density of neurons expressing Fosab protein in the telencephalon of 5 dpf larvae exposed to DMSO ($N = 3$; $n = 8$) and 15 µM DFP ($N = 3$; $n = 8$) (unpaired *t*-test: ****, $P < 0.0001$). N = number of larvae and n = number of slices. Abbreviation: Tel, telencephalon.

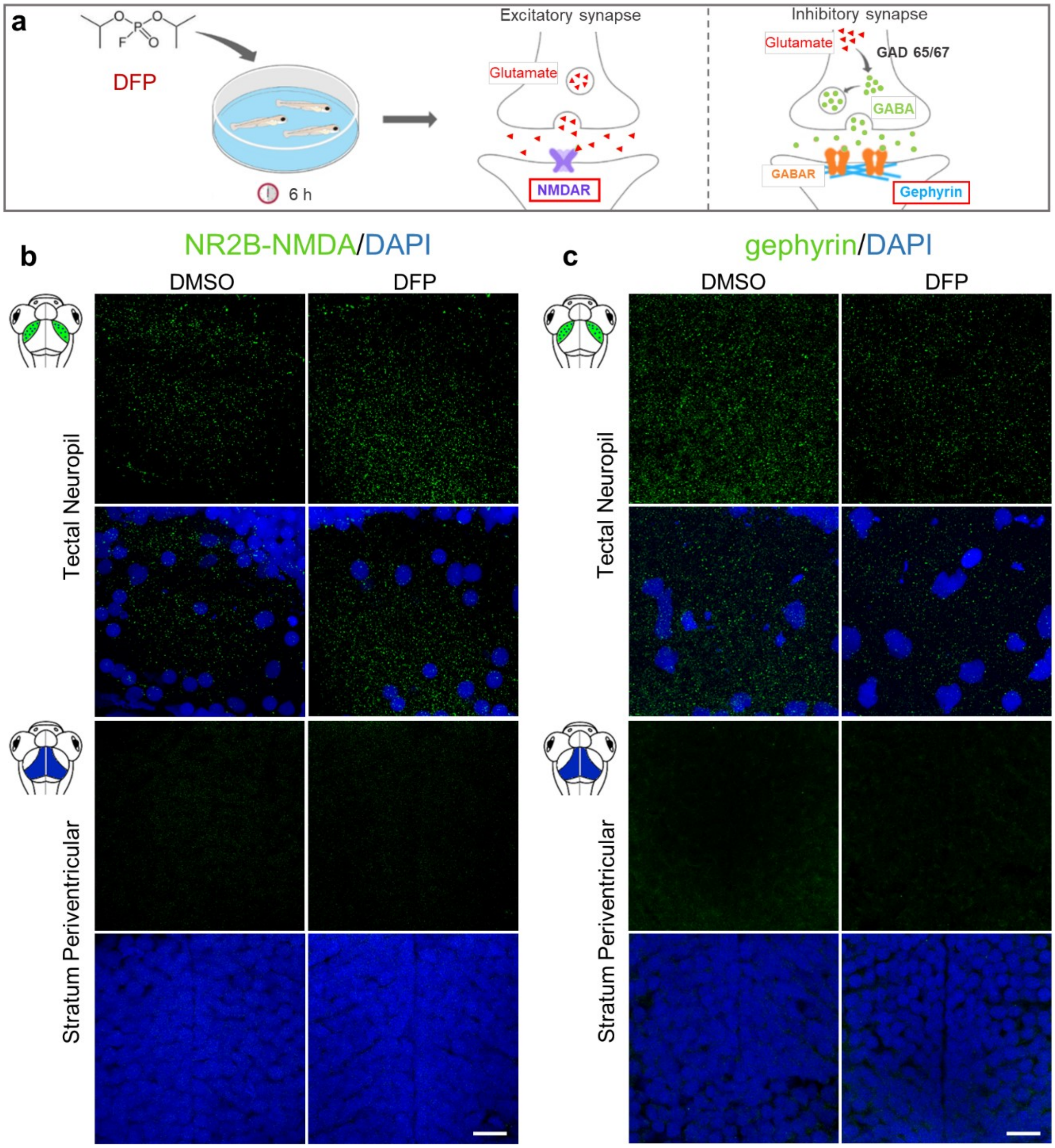

Supplementary Fig. 4 NR2B-NMDA and gephyrin immunostaining. (**a**) As experimental set-up, 5 dpf larvae were exposed to either 15 µM DFP or vehicle (DMSO) for 6 hours, prior to being processed for NR2B-NMDA or gephyrin immunolabeling. (**b**) NR2B-NMDA immunolabeling of 5 dpf larvae brains exposed to either DMSO or 15 µM DFP, showing specific protein accumulation in the tectal neuropils and its absence around cell bodies in the stratum periventricular. Scale bar: 10 µm. (**c**) Gephyrin immunolabeling of 5 dpf larvae brains exposed to either DMSO or 15 µM DFP, showing specific protein accumulation in the tectal neuropils and its absence around cell bodies in the stratum periventricular. Scale bar: 10 µm.

<u>Supplementary video</u>

Supplementary Video 1. 3 minute-long representative recording of calcium activity imaging in optic tectum neurons of 5 dpf larvae following 3 h exposure to vehicle (DMSO). Movie played at 25 fps.

Supplementary Video 2. 3 minute-long representative recording of calcium activity imaging in optic tectum neurons of 5 dpf larvae following 3 h exposure to 15 μM DFP. Movie played at 25 fps.
